# Regional structural impairments outside lesions are associated with verbal short-term memory deficits in chronic subcortical stroke

**DOI:** 10.18632/oncotarget.15882

**Published:** 2017-03-03

**Authors:** Qingqing Diao, Jingchun Liu, Caihong Wang, Jingliang Cheng, Tong Han, Xuejun Zhang

**Affiliations:** ^1^ Department of Radiology and Tianjin Key Laboratory of Functional Imaging, Tianjin Medical University General Hospital, Tianjin 300052, China; ^2^ Department of Radiology, The First Affiliated Hospital of Zhengzhou University, Zhengzhou 450052, China; ^3^ Department of Radiology, Tianjin Huanhu Hospital, Tianjin 300350, China; ^4^ School of Medical Imaging, Tianjin Medical University, Tianjin 300070, China

**Keywords:** ischemic stroke, verbal short-term memory, grey matter volume, voxel-based lesion symptom mapping

## Abstract

**Background and Purpose:**

We aimed to explore the neural mechanisms of verbal short-term memory (VSTM) impairment in subcortical stroke by evaluating the contributions of lesion and remote grey matter volume (GMV) reduction.

**Results:**

There was no significant correlation between lesions and VSTM. In stroke patients with left lesions, GMV reductions in the right middle frontal gyrus and in the left inferior frontal gyrus were positively correlated with VSTM impairment. In patients with right lesions, GMV reduction in the right dorsal posterior cingulate cortex was positively correlated with VSTM impairment.

**Materials and Methods:**

Ninety-seven patients with chronic subcortical ischemic stroke and seventy-nine healthy controls underwent VSTM and structural MRI examinations. Voxel-based lesion symptom mapping was used to identify correlations between lesions and VSTM. Voxel-wise comparisons were used to identify brain regions with significant GMV reduction in patients with left and right lesions. These regions were used in correlation analyses between GMV and VSTM in each patient subgroup.

**Conclusions:**

These findings suggest that VSTM impairment in subcortical stroke is associated with secondary regional structural damage in non-lesion regions, rather than with the lesion itself. Moreover, different neural substrates may underlie VSTM impairment in stroke patients with left and right lesions.

## INTRODUCTION

Stroke affects millions of people around the world annually. In addition to motor deficit, a considerable number of stroke patients suffer from memory impairment that affect their quality of life and recovery in the chronic phase [1]. Cognitive decline in multiple domains, including memory impairment, has been observed in cortical stroke, and the severity of cognitive decline depends on the volume and location of lesions [2, 3]. Memory deficits have also been observed in patients with subcortical stroke [4–6], who exhibit even more severe verbal memory impairment than patients with cortical lesions [7]. Many studies have focused on long-term episodic memory impairment after stroke and have associated it with structural and functional damage in the hippocampus or medial temporal lobe [8–10]. Although verbal short-term memory (VSTM) impairment has been frequently reported in stroke patients and even in patients without long-term memory deficit [11, 12], the neural mechanisms underlying VSTM impairment after stroke remain largely unknown.

Direct structural damage by stroke lesions may be one possible neural mechanism for VSTM impairment. For example, a pioneer study has shown that poor VSTM can be predicted by lesion characteristics: patients with left hemispheric, subcortical, and large lesions showed poor memory performance [7]. In contrast to considering the lesion location and volume as independent predictors, a prior study has proposed combining lesion location and volume to evaluate the clinical outcome after stroke [13]. For example, voxel-based lesion-symptom mapping (VLSM) has been used to investigate the relationship between stroke lesions and VSTM performance, and the results revealed that VSTM impairment is related to lesions in the thalami, the left medial temporal and temporooccipital structures, the right hippocampus and the inferior frontal gyrus [14]. However, those studies included mixed (cortical and subcortical) stroke patients, leaving the exact association between subcortical stroke lesions and VSTM impairment still unknown. In addition to the lesion itself, stroke lesion may lead to impairments in remote regions via the mechanism of axonal degeneration [15, 16]. Identifying the association between remote structural damage and VSTM impairment may improve our understanding of the neural mechanisms underlying VSTM impairment in subcortical stroke patients.

In this study, we aimed to investigate the neural mechanisms underlying VSTM impairment in subcortical stroke patients from two perspectives: (1) testing the effect of the lesion itself by using VLSM to investigate the association between subcortical stroke lesions and VSTM impairment, and (2) testing the effect of remote impairment by assessing the association between structural damage in non-lesion regions and VSTM impairment. Because left and right subcortical stroke lesions may result in different remote structural impairments, we investigated the association between remote structural impairments and VSTM deficits in subcortical stroke patients with lesions in the left and right hemispheres, respectively.

## RESULTS

### Demographic, clinical and behavioral data

The demographic, clinical and behavioral data on stroke patients and healthy controls are shown in Table 1. Forty-nine patients had stroke lesions in the left hemisphere, and 48 patients had stroke lesions in the right hemisphere. No significant differences were found regarding age (*P* = 0.79) among the left-sided stroke patients, right-sided stroke patients and healthy controls in one-way ANOVA. No significant differences were found regarding sex (*P* = 0.15) among the left-sided stroke patients, right-sided stroke patients and healthy controls by the Chi-square test. Patients with lesions in the left (*P* < 0.001) and right (*P* < 0.001) hemispheres both showed worse performance in VSTM examination than the healthy controls.

**Table 1:** Demographic, clinical information and verbal short-term memory performance of stroke patients and healthy controls

Variables	Left-sided Stroke (*n* = 49)	Right-sided Stroke (*n* = 48)	Healthy Controls (*n* = 79)
Age (years)	55.9 ± 7.6 (43–75)	56.1 ± 7.4 (40–71)	55.3 ± 7.3 (40–72)
Sex (male/female)	39/10	33/15	50/29
Duration (months)	20.0 ± 12.5 (6–69)	16.8 ± 8.9 (6–53)	
Verbal short-term memory	42.6 ± 8.2 (28–64)	42.6 ± 10.2 (17–60)	49.9 ± 8.1 (29–73)

Data are presented as mean ± SD (range).

### Voxel-based lesion-symptom mapping

**Figure 1:**
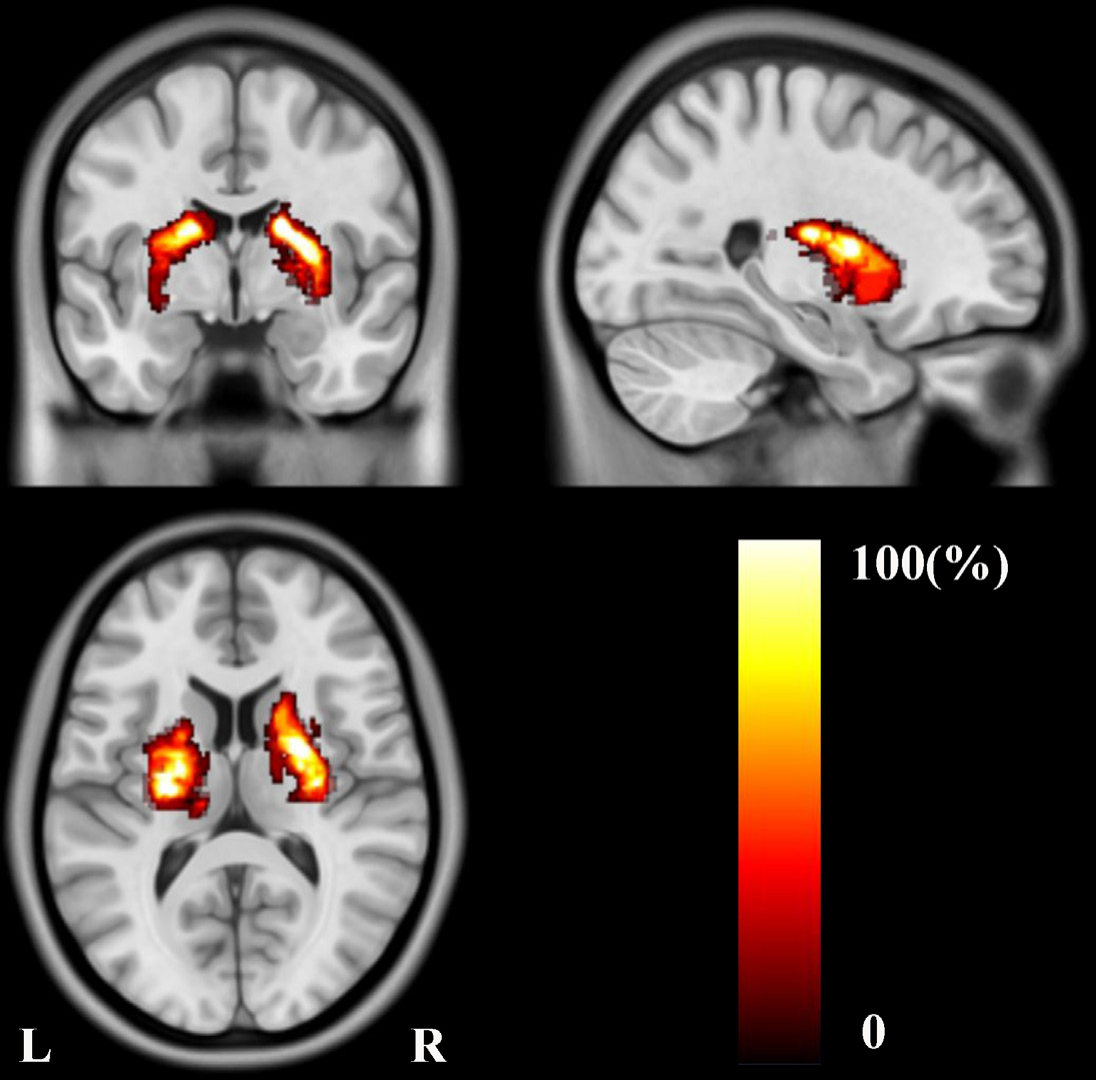
Lesion incidence map of patients with stroke

The lesion incidence map of the stroke patients is displayed in Figure 1. Stroke lesions were primarily distributed in the basal ganglia, internal capsule, thalamus, and corona radiate. The VLSM analysis showed no significant association between lesion location and VSTM when age, sex and lesion volume were included as covariates. There was likewise no significant correlation between lesion volume and VSTM.

### GMV analyses

**Figure 2:**
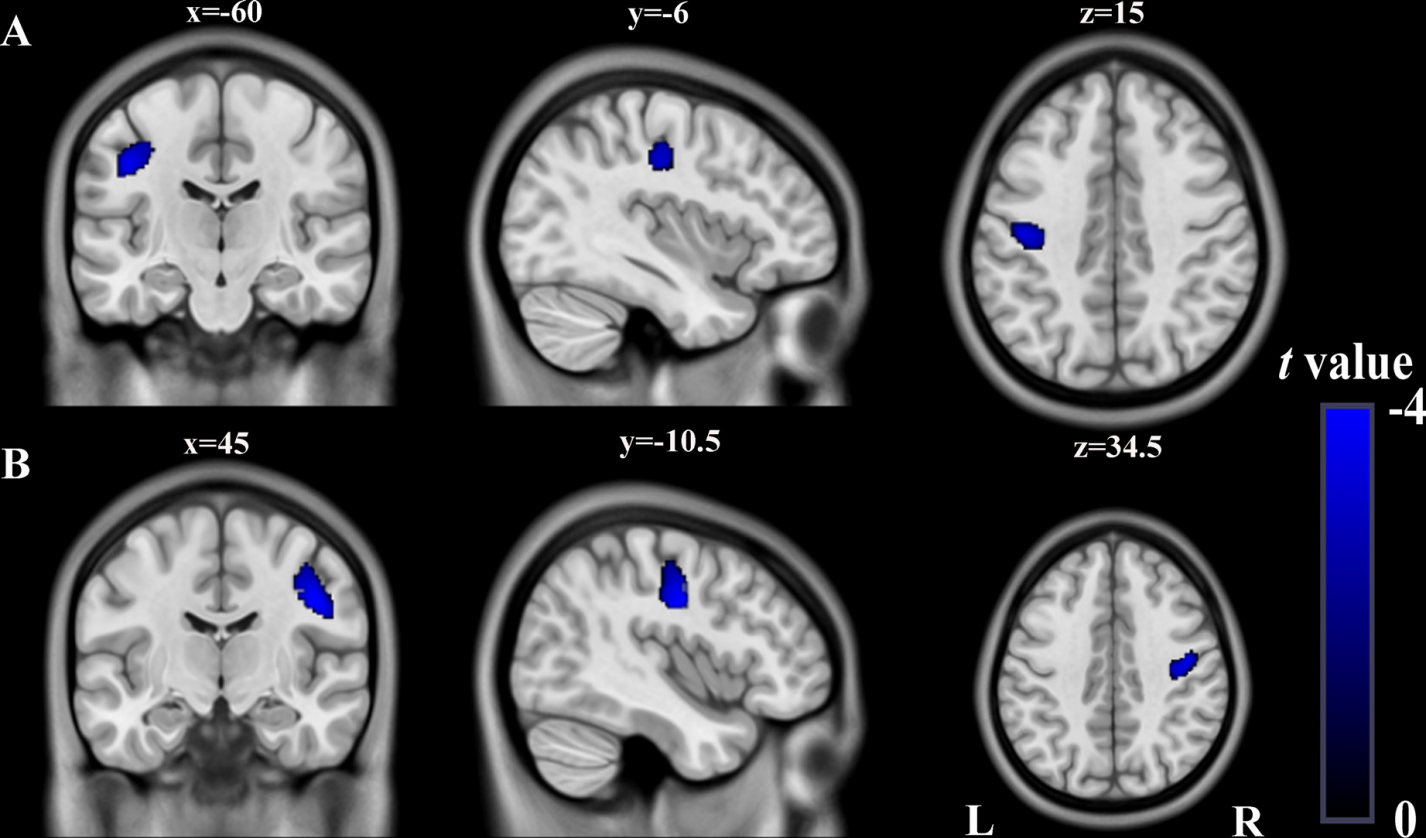
Significant GMV decrease in patients with left- and right-sided lesions compared to healthy controls (*P* < 0.05, FWE-corrected at cluster-level combined with uncorrected *P* < 0.005) (**A**) Stroke patients with left-sided lesions exhibit decreased GMV in L_SMC compared to healthy controls (cluster size>1097). (**B**) Stroke patients with right-sided lesions exhibit decreased GMV in R_SMC compared to healthy controls (cluster size>1107). Abbreviations: FWE, family-wise error; GMV, grey matter volume; L, left; R, right; SMC, sensorimotor cortex.

**Figure 3:**
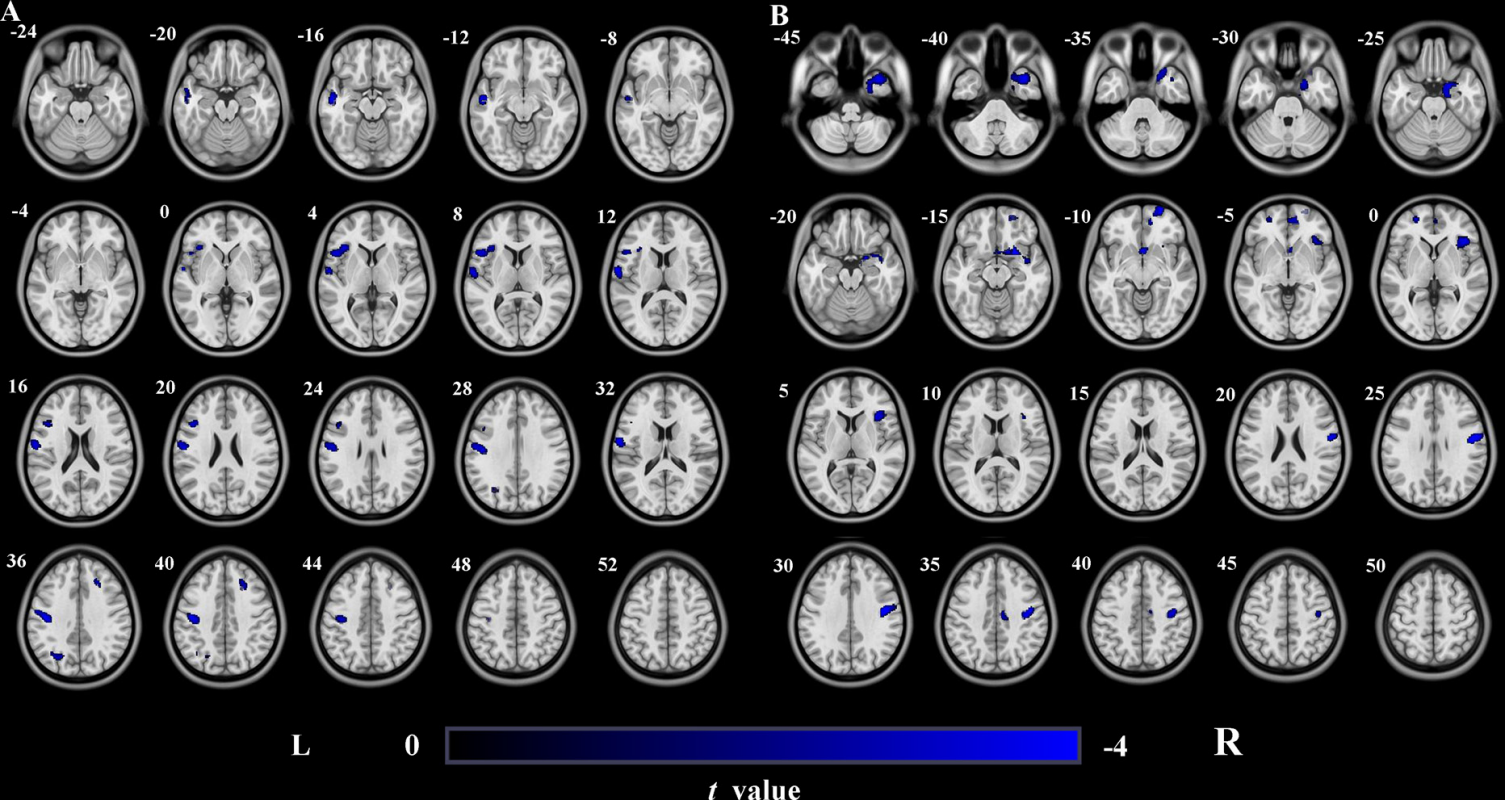
GMV decrease in patients with left- and right-sided lesions compared to healthy controls (uncorrected *P* < 0.01, cluster size>100) (**A**) Stroke patients with left-sided lesions exhibit decreased GMV in L_Ins, L_MTG, L_SMC, R_MFG, L_MOG and triangular part of L_IFG compared to healthy controls. (**B**) Stroke patients with right-sided lesions exhibit decreased GMV in R_Ins, R_MTG, R_SMC, R_dPCC, R_OFC and L_SFG compared to healthy controls. Abbreviations: d, dorsal; GMV, grey matter volume; IFG, inferior frontal gyrus; Ins, Insula; L, left; MFG, middle frontal gyrus; MOG, middle occipital gyrus; MTG, middle temporal gyrus; OFC, orbitofrontal cortex; PCC, posterior cingulated cortex; R, right; SFG, superior frontal gyrus; SMC, sensorimotor cortex.

Using a strict statistical threshold (*P* < 0.05, FWE cluster-level correction combined with uncorrected *P* < 0.005), compared to the healthy controls, stroke patients with left lesions exhibited significantly decreased GMV in the left sensorimotor cortex (SMC) (Figure 2A), and patients with right lesions showed significantly decreased GMV in the right SMC (Figure 2B). Under a loose statistical threshold (*P* < 0.01, uncorrected), patients with left lesions additionally showed decreased GMV in the left insular cortex, middle temporal cortex, middle occipital cortex, triangular part of the inferior frontal gyrus (IFG), and right middle frontal gyrus (MFG) (Figure 3A); patients with right lesions also showed decreased GMV in the right insular cortex, middle temporal cortex, dorsal posterior cingulate cortex (PCC), orbitofrontal cortex, and left superior frontal cortex (Figure 3B).

### Correlation analyses

**Figure 4:**
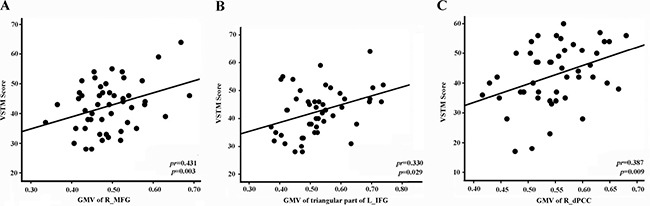
Correlations between GMV values and VSTM scores in stroke patients (**A**) Stroke patients with left-sided lesions exhibit correlations with VSTM score in R_MFG. (**B**) Stroke patients with left-sided lesions exhibit correlations with VSTM score in triangular part of L_IFG. (**C**) Stroke patients with right-sided lesions exhibit correlations with VSTM score in R_dPCC. Abbreviations: d, dorsal; GMV, grey matter volume; IFG, inferior frontal gyrus; L, left; MFG, middle frontal gyrus; PCC, posterior cingulated cortex; R, right; VSTM, verbal short-term memory.

In stroke patients with left lesions, the GMVs in the right MFG (*pr* = 0.431; *P* = 0.003; Figure 4A) and in the left triangular part of the IFG (*pr* = 0.330, *P* = 0.029; Figure 4B) were both positively correlated with VSTM. In stroke patients with right lesions, however, only the GMV in the right dorsal PCC (*pr* = 0.387, *P* = 0.009; Figure 4C) was positively correlated with VSTM.

## DISCUSSION

VSTM reflects the ability to learn and to remember, which is critical for routine human life. Consistently with previous findings [7, 17, 18], we found that chronic subcortical stroke patients with lesions in the left and right hemispheres both showed VSTM deficits. These findings suggest that there is no laterality preference of subcortical stroke lesions in VSTM impairment. There may be two possible neural mechanisms underlying VSTM impairment from the perspective of structural damage: direct structural damage by stroke lesions and secondary atrophy in remote regions caused by stroke lesions via axonal degeneration.

First, the VLSM analysis did not reveal any significant association between lesion location and VSTM impairment, and the correlation analysis between lesion volume and VSTM impairment did not reveal any significant correlation, which is consistent with the results of a previous study [4]. These findings suggest that lesion damage itself may not be the critical neural mechanism for VSTM impairment in subcortical stroke patients. These findings contrast with previous studies including patients with both cortical and subcortical stroke, which frequently report associations between cognitive decline and lesion location and volume [7, 19]. Taken together, these conflicting results may indicate that cortical damage is critically important for VSTM deficits in stroke patients.

Another possible neural mechanism underlying VSTM deficits in patients with subcortical stroke is structural damage in remote regions, caused by axonal degeneration secondary to stroke lesions [15, 16]. Consistently with previous studies investigating GMV changes in subcortical stroke [20, 21], we found that the ipsilesional SMC was the brain region with the most significant GMV reduction. However, we found no significant correlation between GMV reduction in this region and VSTM impairment in these stroke patients, suggesting that structural damage in the SMC is not important for VSTM impairment in subcortical stroke. On the contrary, these mildly structurally impaired brain regions may compromise VSTM impairment in subcortical stroke patients, in which only a small part of fibers are impaired by stroke lesions. Specifically, VSTM impairment was correlated with the GMV in the right MFG and the left triangular part of the IFG in patients with left lesions and in the right anterior part of the dorsal PCC in patients with right lesions.

As a subset of working memory, VSTM performance may also depend on the structural and functional integrity of two anti-correlated networks: the working memory network and the default mode network (DMN). The working memory network includes a set of brain regions, mainly the MFG, IFG and superior parietal lobe (e.g., [22–25]). This set may explain our finding that GMV reduction in the MFG and IFG was correlated with VSTM impairment in stroke patients. Because cognitive performance depends on the balance between cognitive network and the DMN, the brain regions of the DMN are also important for cognitive processing. As a critical brain region of the DMN, the dorsal PCC is also associated with working memory performance [26]. This association may explain why GMV reduction in the dorsal PCC was correlated with VSTM impairment in stroke patients. Because lesion locations are not completely consistent between the two hemispheres, the impaired regions and connections are also different between the hemispheres. These differences may be related to the different neural mechanisms of VSTM impairment in chronic subcortical stroke patients with lesions in the left and right hemispheres.

In conclusion, we found that VSTM impairment in subcortical stroke is associated with secondary regional structural damage in the non-lesion regions, rather than the lesion itself. This result suggests that secondary regional atrophy contributes to VSTM impairment and may be a predictor of VSTM performance in subcortical stroke patients, providing a novel perspective for further study. Moreover, the brain regions correlated with VSTM impairment in stroke patients with left and right lesions were different, suggesting that different neural substrates may underlie VSTM impairment. Thus, the lesion-side effect should be considered in future studies on cognitive function following stroke.

## MATERIALS AND METHODS

### Subjects

The subjects were recruited from three medical centers: Tianjin Medical University General Hospital, Tianjin Huanhu Hospital and the First Affiliated Hospital of Zhengzhou University. The study protocol was approved by the Medical Research Ethics Committees of the hospitals, and all subjects provided written informed consent before examination. The inclusion criteria for patients were as follows: (1) first-onset ischemic stroke; (2) a single lesion located in the internal capsule and neighboring regions; (3) right-handed before the stroke; and (4) time after stroke onset > 6 months. The exclusion criteria for patients were as follows: (1) recurrent stroke after first onset; (2) any other brain abnormalities; (3) unable to complete the memory test; (4) severe white matter hyperintensity manifesting as a Fazekas et al [27] scale score of > 1; and (5) a history of drug dependency or psychiatric disorders. A total of 97 patients (72 men and 25 women; mean age, 56.1 ± 7.5 years) were included in this study according to the above criteria. Seventy-nine healthy subjects (50 men and 29 women; mean age, 55.3 ± 7.3 years) were also recruited as controls. These participants were recruited from Tianjin Medical University General Hospital (33 patients, 25 healthy controls), Tianjin Huanhu Hospital (29 patients, 25 healthy controls) and the First Affiliated Hospital of Zhengzhou University (35 patients, 29 healthy controls).

### MR data acquisition

Three-dimensional sagittal T1-weighted images were acquired using three 3.0-Tesla MR scanners from the three hospitals, including two Discovery MR750 scanners (General Electric, Milwaukee, WI, USA) and a Magnetom Trio Tim MR scanner (Siemens, Erlangen, Germany). The repetition time (ms)/echo time (ms)/flip angle/matrix/slices were 8.2/3.2/11°/256 × 256/188 for the MR750 scanner and 2000/2.3/9°/256 × 232/192 for the Trio Tim scanner. All scans used the same field of view (256 mm × 256 mm), slice thickness (1 mm and no gap) and spatial resolution (1 × 1 × 1 mm^3^).

### Verbal short-term memory assessment

We used the Rey Auditory Verbal Learning Test (RAVLT) to evaluate the VSTM function. The RAVLT is a widely used test for assessing verbal memory performance and shows good reliability and validity [7, 28–30]. This test consists of 5 trials. In each trial, the examiner read a list of 15 words loudly at the speed of one word every two seconds, and the participants were told to listen carefully. After the presentation, the participants were asked to recall as many of the presented words as possible. The total number of correctly recalled words in all 5 trials was recorded as the VSTM score.

### GMV calculation

The GMV maps were calculated using the VBM8 implemented in the Statistical Parametric Mapping software package (SPM8, http://www.fil.ion.ucl.ac.uk/spm). The structural MR images were segmented into grey matter (GM), white matter and cerebrospinal fluid using the VBM8 segmentation, which can estimate the fraction of each pure tissue type present in every voxel. The individual's GM concentration map was then normalized to the Dartel template in Montreal Neurological Institute (MNI) space (http://www.mni.mcgill.ca/). This template was derived from 550 healthy control subjects in the IXI-database (http://www.brain-development.org). In the modulated normalized process, we multiplied the individual's GM concentration map only by the non-linear determinants derived from the spatial normalization procedure [31]. This step resulted in a normalized GM density or relative GMV map for each subject. Here, the GMV of each voxel represents the fraction of GM present in each voxel, which preserves the local GM density while removing the confounding effect of variance in individual brain size. Next, we resliced the normalized GMV to a 1.5-mm cubic voxel. Finally, the GMV images were smoothed with a kernel of 8 × 8 × 8 mm^3^ full width at half maximum. Then, the spatial pre-processing, normalized, modulated, and smoothed GMV maps were used for further analysis.

### Voxel-based lesion-symptom mapping

Voxel-based lesion symptom mapping (VLSM) was used to test the association between VSTM and lesions [14, 32–36]. After normalizing the 3D T1-weighted images to MNI space [35], the stroke lesions were blindly outlined by three radiologists on the individual's T1-weighted images (reference to T2-weighted images) using the MRIcron software (https://www.nitrc.org/projects/mricron). Then, we checked the consistency of lesion volume across the three experimenters and found the intra-class correlation coefficient to be 98%, so we selected the lesion maps outlined by the senior radiologist for the next process. We used VLSM version 2.55 (https://langneurosci.mc.vanderbilt.edu/resources.html) [37] to investigate the correlation between lesion location and VSTM score after controlling for age, sex, and lesion volume. Statistical significance was determined using a voxel-wise threshold of *P* < 0.01 and a cluster-wise threshold of *P* < 0.05 based on permutation testing. In addition, the association between lesion volume and VLSM score was examined using partial correlation analysis with age and sex as covariates of no interest (*P* < 0.05).

### GMV analyses

For each patient subgroup, a two-sample *t*-test was used to compare GMV differences between patients and controls in a voxel-wise manner with age, sex and scanner as covariates of no interest. We used two thresholds to identify the brain regions with GMV reduction in the stroke patients. A family-wise error (FWE) correction for multiple comparisons (*P* < 0.05, cluster-level correction combined with uncorrected *P* < 0.005) was used to identify brain regions with significant GMV reduction in stroke patients. In addition, a loose threshold (*P* < 0.01, uncorrected) was used to identify brain regions with mild GMV reduction. These two types of brain regions were extracted as regions of interest (ROIs) and used for ROI-based analyses.

### Correlation analyses

A partial correlation analysis (*P* < 0.05, uncorrected) was performed to test for correlations between the GMVs of these ROIs and VSTM in stroke patients, controlling for age, sex and scanner.
